# Prescriptions and Dispensing

**Published:** 1908-11-07

**Authors:** 


### PRESCRIPTIONS AND DISPENSING.

### COMPRESSED TABLETS.

Compressed Tablets have come to be regarded too much as solely the product of wholesale manufacturers. It is not only possible to make tablets with a comparatively simple hand-machine in private practice, but it is also a considerable saving in expense if tablets are to be used to any great extent. They are much cheaper to make than to buy, once the machine is in working order.

The tablet occupies in some respects an intermediate position between the powder and the pill. If, for instance, sulphonal is given in tablet form, it is essential that within a very short time after taking it the drug shall be in the same condition in the stomach as it would be if it had been swallowed in a cachet. The tablet form is prescribed chiefly because of its compactness and portability, but partly also because some patients prefer a tablet to a powder or a cachet. The tablet here approximates very closely to a form of powder, and the principal requirement is that it shall become a powder very readily. This is by no means a character always complied with by tablets manufactured wholesale, particularly when they have been extremely compressed by steam power; it often happens that such tablets are passed unaltered by the patient.

Tablets may also be prescribed in place of pills when they contain extracts, etc.; it is sometimes possible to ensure dissolution of the tablet more quickly than that of a corresponding pill.

Other tablets, again, are strictly lozenges, and their hardness and slow solubility are then the qualities chiefly to be aimed at.

The special further requirements needed by all tablets are perfect uniformity of the material, the greatest possible uniformity in the weight of the individual tablets, and the best possible appearance or finish.

There is far more divergence in the patterns of tablet machines for dispensing than is to be found among pill machines. They agree, however, in the essential parts, which are—(1) an upper and lower punch, usually of steel, between which the material is compressed; (2) an eye, or small cylinder of steel bored with a hole just large enough in diameter for the punches to move freely in it; this cylinder is both the measure into which the exact amount of material for one tablet is measured, and also the chamber in which the actual compression takes place; (3) a feeder, consisting of a hopper, in which the material is placed, and by which it is supplied to the eye; by a simple arrangement the foot of the hopper is usually made to push aside the tablet made at the previous stroke, before giving a fresh supply of material for the next.

The force of the stroke in a hand machine is sometimes given directly by forcing down a lever; in others indirectly by giving another lever a to-and-fro motion; the latter plan is the better calculated to secure uniformity of pressure, and therefore of hardness in the products.

With a little practice, regularity in working the machine is easily attained, and most of the skill of tablet-making is required in preparing the material for compression. We shall first consider the requirements which are common to all materials before discussing special difficulties.

The machine is adjusted for producing tablets of different weights by the use of eyes and punches of different diameters, and also by raising or lowering the lower punch in the eye until the latter, when filled to the top by the feeder, holds exactly the weight of material required in one tablet. These adjustments having been made, the weights of the individual tablets in a batch will depend upon the particles of the material in the hopper being uniform in size, and in such a condition that they will run easily through the opening in the foot of the hopper. Fine powders will not run easily, and they should not be used; a granular powder, the particles of which will just pass a No. 20 brass sieve, is usually the best. If there is much fine powder with the granules it will sift to the bottom of the hopper with the vibration, and since it will lie closer than the coarser granules, the tablets produced at first will weigh considerably more than those which follow. If, on the other hand, the granules are too coarse, the quantity that fills the eye will vary from time to time, and some of the tablets will be too light.

All that is necessary for the production of satisfactory granules in the simplest cases is to coarsely powder the material. Potassium bromide may be taken as an example of the substances that can be dealt with in this way. The salt is triturated in a mortar and shaken through a sieve of No. 20 mesh. The coarser pieces which do not pass through are again rubbed down. The whole should be transferred to the sieve at short intervals, so that the particles that are small enough to pass shall not be crushed still smaller. It is inevitable, however, that some finer powder should be produced, and when all the salt has passed the No. 20 sieve it must be put in a rather finer one, about No. 30 being best, and all that will pass this finer sieve is rejected so far as the tablet-making is concerned. It may be used for other purposes. The remainder will now be in very nearly uniform particles. If not perfectly dry, as is probable owing to there being traces of moisture enclosed in the original large crystals, it should be dried for a short time, and it is then ready for compressing without further addition or treatment. The granules will run easily, and the amount that fills the eye of the machine each time will be practically constant. Many soluble salts, though not all, can be prepared in a similar way.

When dealing with a soluble substance it is not as a rule necessary to add anything to assist in disintegrating it, and it is undesirable to add anything which will prevent the formation of a clear bright solution. Many of the substances, however, that are given in tablet form sometimes, phenacetin and sulphonal for example, are not soluble in aqueous liquids; it is therefore necessary to add something which will assist the disintegration of the tablets, and such a material is found in starch.

(*To be continued.*)

